# Direct Imaging of RAB27B-Enriched Secretory Vesicle Biogenesis in Lacrimal Acinar Cells Reveals Origins on a Nascent Vesicle Budding Site

**DOI:** 10.1371/journal.pone.0031789

**Published:** 2012-02-20

**Authors:** Lilian Chiang, Serhan Karvar, Sarah F. Hamm-Alvarez

**Affiliations:** 1 Pharmacology and Pharmaceutical Sciences, School of Pharmacy, University of Southern California, Los Angeles, California, United State of America; 2 Medicine, Keck School of Medicine, University of Southern California, Los Angeles, California, United States of America; 3 Physiology and Biophysics, Keck School of Medicine, University of Southern California, Los Angeles, California, United States of America; Purdue University, United States of America

## Abstract

This study uses YFP-tagged Rab27b expression in rabbit lacrimal gland acinar cells, which are polarized secretory epithelial cells, to characterize early stages of secretory vesicle trafficking. Here we demonstrate the utility of YFP-Rab27b to delineate new perspectives on the mechanisms of early vesicle biogenesis in lacrimal gland acinar cells, where information is significantly limited. Protocols were developed to deplete the mature YFP-Rab27b-enriched secretory vesicle pool in the subapical region of the cell, and confocal fluorescence microscopy was used to track vesicle replenishment. This analysis revealed a basally-localized organelle, which we termed the “nascent vesicle site,” from which nascent vesicles appeared to emerge. Subapical vesicular YFP-Rab27b was co-localized with p150^Glued^, a component of the dynactin cofactor of cytoplasmic dynein. Treatment with the microtubule-targeted agent, nocodazole, did not affect release of mature secretory vesicles, although during vesicle repletion it significantly altered nascent YFP-Rab27b-enriched secretory vesicle localization. Instead of moving to the subapical region, these vesicles were trapped at the nascent vesicle site which was adjacent to, if not a sub-compartment of, the trans-Golgi network. Finally, YFP-Rab27b-enriched secretory vesicles which reached the subapical cytoplasm appeared to acquire the actin-based motor protein, Myosin 5C. Our findings show that Rab27b enrichment occurs early in secretory vesicle formation, that secretory vesicles bud from a visually discernable nascent vesicle site, and that transport from the nascent vesicle site to the subapical region requires intact microtubules.

## Introduction

Apically-secreting epithelial cells of the lacrimal gland are organized around lumina continuous with tear ducts which drain contents on to the ocular surface. Inside these lacrimal gland acinar cells (LGAC), vital tear fluid and proteins, including antibacterial and antiviral factors like secretory IgA [1] and proteases [2], as well as mitogenic proteins such as lacritin [3] and EGF [4], are packaged into secretory vesicles (SV). Intracellular transport of these SV involves three main steps: vesicle formation, maturation, and fusion with the apical plasma membrane. In secretory epithelial cells, SV maturation is marked by changes in SV size [5], [6], SV density and content [7], [8], and the recruitment of proteins, such as Rab3D, to the surface of the SV membrane [9]. Secretory epithelial cells respond to specific agonists which accelerate the final fusion of mature SV with the apical membrane, causing the release of SV contents into the lumen. Studies in acinar cells have described the accumulation of mature SV in the subapical region of the cells in preparation for this fusion event [6], [10], [11], which likely occurs in conjunction with homotypic fusion [12] and in parallel with membrane recycling [13], [14]. While many questions remain regarding the mechanisms that must take place for SV maturation and fusion, SV formation and their early transport from the site of origin is even less well-understood. Classical studies of transport vesicle budding in professional secretory cells suggest that SV budding and fission occur in the basolaterally-organized Golgi stacks and trans-Golgi network (TGN) [15], [16], [17], but much of this data is based on static techniques such as electron microscopy. Studies have been limited both temporally and by the scarcity of early SV-specific markers which are necessary to differentiate the early SV from TGN or another non-SV material.

Factors implicated so far in acinar SV trafficking include the microtubule and actin networks. In LGAC, the minus-ends of microtubules are organized beneath the apical plasma membrane, allowing polarized and apically-targeted cytoskeletal-based cargo transport, such as that facilitated by the minus-end directed cytoplasmic dynein motor, to occur [13], [18]. Cytoplasmic dynein, itself a large multi-subunit protein complex, associates with a multiprotein accessory complex known as dynactin which includes the polypeptide, p150^Glued^ [19]. Once cargo reaches the subapical cytoplasm, studies in diverse epithelial cells suggest a “hand off” from factors which tether the SV to microtubules, to those which tether to actin filaments [13], [20]. Previous studies in LGAC suggest that cytoplasmic dynein and the dynactin complex participate in the stimulated trafficking of SV into the subapical cytoplasm [9]. However, the role of dynein prior to SV maturation and the enrichment of these nascent vesicles with more recently discovered SV protein markers, such as rabs and myosin motors, are not clear.

Actin filaments in LGAC are abundant in the subapical region and are thought to facilitate the fusion of SV to the apical plasma membrane [5]. One superfamily of actin motor proteins, class V myosins (Myosin 5A, Myosin 5B, and Myosin 5C (M5C)), play a role in the tethering and transport of SV in a variety of cells [21], [22], [23], [24]. In particular, M5C is expressed highly in exocrine secretory tissues and has been shown to be associated with mature SV in LGAC and to participate in apical SV exocytosis [6], [25].

The Rab family is comprised of over 60 different monomeric proteins which are highly involved in membrane trafficking [26], [27]. Regulated binding and hydrolysis of GTP by the Rabs is coupled to membrane association and disassociation, a process which in turn is linked to the regulation of membrane sorting and fusion events [28], [29], [30]. In professional secretory cells, studies have largely focused on the role of Rab3 isoforms in exocytosis. Rab3A, Rab3B, and Rab3C isoforms are highly expressed in endocrine and brain tissues and participate primarily in terminal exocytotic events [31]. In acinar cells from different exocrine tissues including LGAC, Rab3D is the most highly expressed Rab3 isoform and participates in terminal exocytosis, which enables its use as a mature SV marker [32], [33], [34], [35].

More recent work in acinar cells has focused on Rab27. While differentially expressed in cells, the Rab27 isoforms, Rab27a and Rab27b, also appear to participate in aspects of exocytosis [36], [37]. In LGAC, Rab27b is highly expressed and is important for SV exocytosis, as evidenced by findings that the overexpression of dominant-negative Rab27b significantly suppressed stimulated exocytosis of SV in pancreatic acini [38], parotid acini [39], [40], and LGAC [41]. Studies in Rab27b knockout mice of platelet cells and LGAC also showed that a loss of Rab27b functionality correlated with a decreased number of dense granules/SV [41], [42]. Rab27b interacts with effectors which mediate interaction with the actin cytoskeleton during transport to the plasma membrane for fusion (MyRIP/Exophilin8/Slac2-c) and with the SNARE protein affiliated granule docking (Granuphilin/Exophilin2/Slp4) which suggest its involvement in the final stages of exocytosis [39], [43]. These studies suggest that Rab27b is at least associated with mature SV in the subapical region, if not a wider SV population.

The current state of knowledge regarding intracellular trafficking in secretory acinar cells of exocrine glands is limited by the fact that relatively few SV markers have been identified which remain clearly associated throughout the stages of SV formation, maturation, and fusion. We demonstrate here that Rab27b associates with nascent SVs at early steps in their formation. Rab27b can be therefore be utilized to investigate SV formation and maturation, in addition to events involving terminal exocytosis.

## Results and Discussion

### Rab27b-enriched SV bud from an unidentified nascent SV site in the basal region

**Figure 1:**
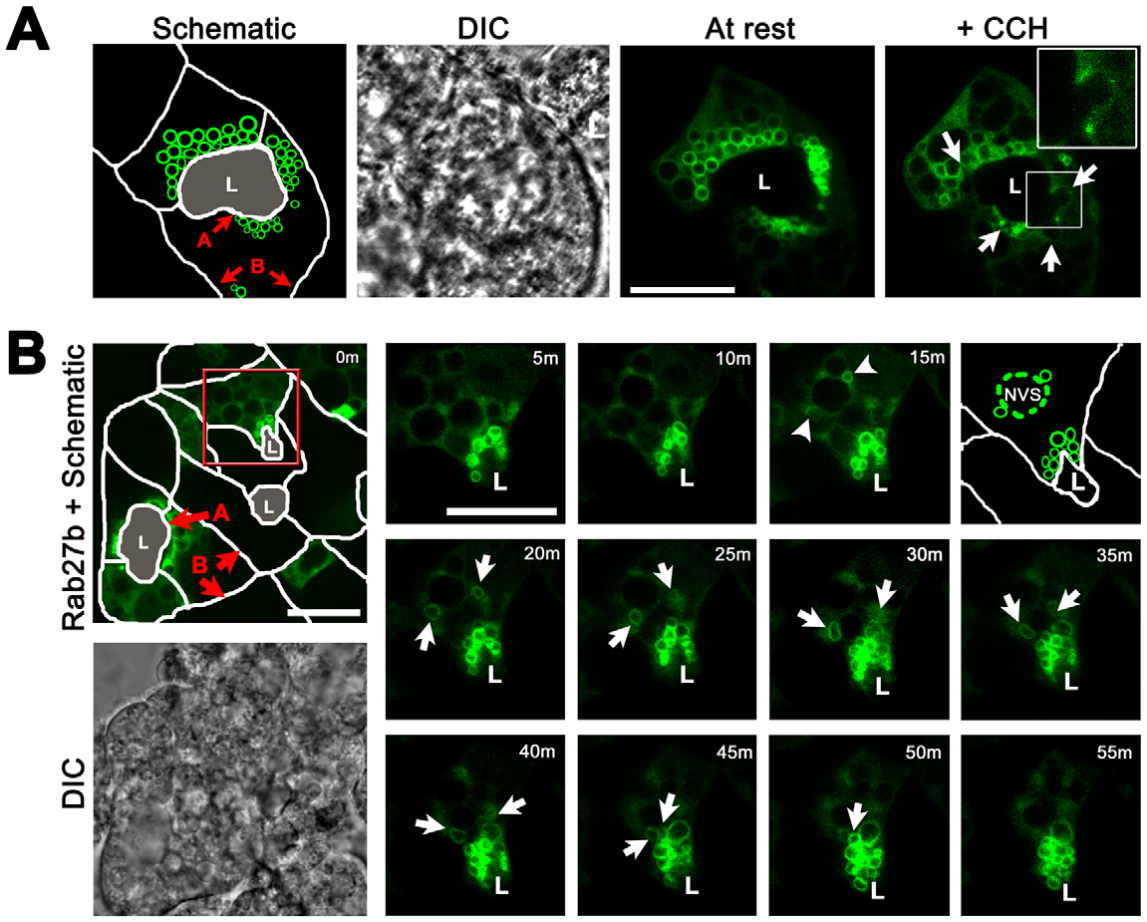
YFP-Rab27b-enriched SV bud from a nascent vesicle site. **A.** YFP-Rab27b expressed in cultured rabbit LGAC was imaged in live cells which exhibited both basolateral (*B*) and apical (*A*) plasma membranes, the latter of which delineated the lumen (*L*). DIC is shown to confirm location of lumena. Image frames show SV expression at rest and with CCH stimulation. *Arrows* represent sites of SV fusion with the apical membrane, while the *inset* shows a higher magnification of a fusion site. Bar represents 10 µm, N = 6. **B.** Schematic depicts orientation of a cluster of LGAC immediately after CCH washout (0 m). The *red box* outlines the region of interest for the remainder of the times-series taken during recovery phase, with representative images taken every 5 m. A schematic at 15 m identifies the supposed NVS from which SV appear to bud (N = 5). *Arrowheads* represent the site of nascent SV emergence; *arrows* follow the movement of these SV to the apical membrane. Bar represents 10 µm.

In culture, LGAC retain their epithelial polarity. Clusters of three or more of these cells reform functional reconstituted acini in vitro, with apical domains organized around luminal regions which are sites of SV exocytosis and which remain open to the culture media. Actin filament labeling patterns, which are abundantly enriched beneath apical and, to a lesser extent, basolateral membranes, can delineate these membrane domains. YFP-tagged Rab27b constructs expressed in cultured LGAC using adenoviral transduction yielded high (70–80%) transduction efficiencies and enabled visualization of vesicular trafficking events. At resting stages in LGAC, a large, subapical SV pool enriched in Rab27b was detected in the subapical region (**Figure 1A**). Upon stimulation with the muscarinic agonist, carbachol (CCH), which simulates the regulated release of SV, this Rab27b-enriched SV population fused with the apical membrane. Fusion was detectable by SV concavities at the apical membrane (*arrows*, in Figure 1A “+CCH” panel and in higher magnification of boxed region). These fusion events have also been described in other works, which have demonstrated that such concavities are accessible to luminal fluid and also interact with actin-coats associated with fusion intermediates [18], [41], [44]. Additionally, use of dominant-negative Rab27b suppressed apical fusion events and reduced secretory protein release in other acinar secretory cell models [38], [39], reinforcing Rab27b as a terminal effector of SV exocytosis in LGAC and other acinar cells. CCH stimulation depleted at least 50% of the YFP-Rab27b associated with the original SV pool after 15m [41]. Exploiting this depletion effect, we established a model which enabled us to follow the replenishment of mature YFP-Rab27b-enriched SV in the subapical region after exocytosis.

LGAC expressing YFP-Rab27b were stimulated with CCH and, after multiple washes, were allowed to reform new SV. A sample time-lapse series of images shown in **Figure 1B** revealed that upon removal of CCH from the cell media, a new pool of SV enriched in YFP-Rab27b re-accumulated in the subapical region after the first 15 m of recovery. Nascent SV contributing to this vesicle pool were formed from a novel organelle located in the mid-basal region of the cell, which we designated the “Nascent Vesicle Site” (NVS, identified in the schematic representing LGAC after 15 m of recovery). This compartment was larger than a typical mature SV (2–4 µm, compared to an approximate 1 µm diameter) [41] and appeared to transiently accumulate or perhaps recruit Rab27b protein to its membrane during the budding process. The resulting structure, detectable by a faint but distinct labeling of YFP-Rab27b, was clearly discernible in three-dimensional reconstructions of LGAC images (see **Video S1**). Over a 60 m recovery phase from CCH stimulation, we observed that the nascent YFP-Rab27b-enriched SV which formed on the NVS (15 m) were transported from the NVS towards the subapical region (55 m) where they joined the remainder of the original vesicle pool. The time-lapse movie of this SV recovery is shown in its entirety in **Video S2**. These time-lapse studies showed that YFP-Rab27b was not only highly expressed on mature SV in the subapical region, but was enriched on immature SV in the basal regions of the LGAC as well. Real-time imaging also captured the budding event of SV from the NVS in the basal region of the cell. While the basal location of the NVS was consistent with that of the TGN, the classical stacked morphology of the TGN was not obvious in these images of the NVS. Furthermore, the apparent recruitment of YFP-Rab27b to the surface of the NVS structure prompted further questions regarding the nature of this apparent organelle.

### Transport of SV from the NVS to the subapical region of LGAC requires an intact microtubule network

**Figure 2:**
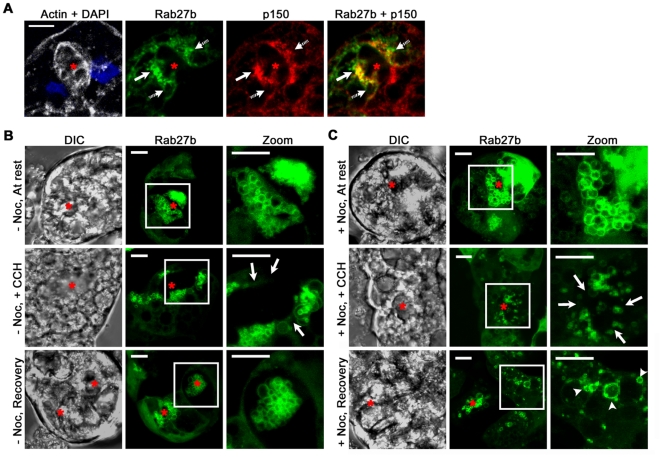
Disruption of the microtubule network hinders nascent YFP-Rab27b-enriched SV production. **A**. Fixed LGAC expressed YFP-Rab27b for direct fluorescence (*green*) was co-detected for endogenous p150^Glued^ (p150; *red*). Regions on the membrane of apparent SV showing strong co-localization are labeled with *solid arrows*, whereas regions of SV expressing only one marker are labeled with *striped arrows*. Actin (*white*, labeled with fluorescent phalloidin); nuclei (*blue*, labeled with DAPI). *Red asterisk* labels the lumen. Bar represents 10 µm, N = 4. **B and C.** Live LGAC expressing YFP-Rab27b were left untreated (**B**) or were treated with 33 µM nocodazole (*Noc*) for 60 m (**C**). Z-stack images were acquired of cells at resting state, 15 m after CCH stimulation, and 60 m after recovery. The outline of the acinus, which corresponds to the flattened z-stack image of YFP-Rab27b (*green*) beside it, can be seen in the DIC image. *Arrows* point to subapical regions with noticeable loss of the SV pool. Insets represent magnifications of the boxed regions; * represents lumen; arrows point to NVS. Bars represent 5 µm, N = 6.

Dynactin and cytoplasmic dynein are thought to be involved in the microtubule-dependent apical targeting of pancreatic zymogen granules [45]. More specifically, the p150^Glued^ subunit of the dynactin complex interacts directly with the cytoplasmic dynein complex and is thought to mediate the binding of dynein to membrane cargoes [46], [47]. In previous work in LGAC, p150^Glued^ expression has been used to infer the distribution of the cytoplasmic dynein complex; these previous studies revealed co-localization of p150^Glued^ with markers of mature SV and further showed that inhibition of p150^Glued^ association with dynein using overexpression of the protein, dynamitin, disrupted the normal maturation of SV [9], [48]. The detection of budding YFP-Rab27b-enriched SV from the NVS provoked further investigation into the mechanisms of SV from the basal NVS to the subapical region. In our initial co-localization study, Rab27b and p150^Glued^ were co-detected in fixed LGAC shown in **Figure 2A**. YFP-tagged Rab27b was partially co-localized (area with high co-localization, *solid arrow*; not co-localized, *striped arrow*) on apparent subapical SV with endogenous p150^Glued^, and had a co-localization coefficient in the subapical region as defined in **Methods** of 55.2%±7.9% (N = 4). These data suggest the involvement of cytoplasmic dynein in the motility of at least a subpopulation of mature SV in the subapical region of acinar cells.

Previous work showed that the disruption of microtubules reduced stimulated lacrimal secretion, likely by affecting earlier stages of SV biogenesis or transport, but not terminal exocytosis [49]. To more directly test the functional role of microtubules and their associated motors like cytoplasmic dynein in Rab27b-enriched SV trafficking, we repeated live-cell imaging studies of the recovery process depicted in **Figure 1B** but with an additional nocodazole treatment which prevented microtubule repolymerization. This dosing regimen resulted in extensive microtubule depolymerization in LGAC and effectively nullified motor-driven microtubule-dependent transport functions [18], [50]. Baseline measurements for fluorescence were first taken in live acini expressing YFP-Rab27b and are represented as three-dimensional z-stack reconstructions in **Figure 2B**. At rest, fluorescence intensity of the total cell area in the subapical region measured 45.8%±7.3% (N = 4). After a pulse of CCH to stimulate exocytosis for 15 m, this signal was significantly decreased to 29.9%±2.1% (p<0.05, N = 4). Allowing for a recovery phase of 60 m following washout of CCH, fluorescence intensity increased significantly compared to the stimulated phase, to 42.5%±8.2% (*P*<0.05, N = 4). Using nocodazole treatment, the effects of microtubule depolymerization on the recovery of Rab27b signal in the subapical region were determined. Nocodazole-treated LGAC reacted similarly to non-treated LGAC during the initial resting and CCH stimulation phases. Results represented by the z-stack reconstructions in **Figure 2C** showed that, similar to non-treated cells, nocodazole-treated LGAC initially expressed a large YFP-Rab27b-enriched SV pool at rest and also retained fusion and release activities upon CCH stimulation (*arrows in zoom images*). Measured signal intensity of subapical YFP-Rab27b fluorescence in these nocodazole-treated LGAC was consistent with that of non-treated cells, with intensity at rest measuring 42.0%±2.6% (N = 4) and decreasing significantly to 31.5%±1.0% with CCH stimulation (*P*<0.05, N = 4). However, unlike non-treated cells, nocodazole-treated LGAC showed loss of the ability to recover the subapical SV pool within the 60 m post-wash phase. This was reflected in a measured signal intensity of 33.2%±2.6% (N = 4), which was not significantly different from the signal intensity measured in stimulated, nocodazole-treated LGAC. Instead, nocodazole-treated LGAC during recovery expressed early-phase SV which appeared unable to bud off completely from the NVS (*arrowheads*), leaving a number of YFP-Rab27b-enriched, small or fragmented SV attached to a faintly Rab27b-enriched membrane reminiscent of the NVS structure detected in **Figure 1B**. This failure to regenerate a SV pool that could replenish the depleted subapical pool is shown in **Video S3**. These observations suggested that intact microtubules are critical to the formation and/or transport of nascent YFP-Rab27b-enriched SV from the NVS to the apical membrane, but not for the final fusion of SV and release of contents. We have previously noted that nocodazole increased punctate labeling of p150^Glued^ at resting phase and also reduced the CCH-induced subapical accumulation of p150^Glued^ [9]. The sequestration of Rab27b-enriched SV coinciding with the impaired accumulation of subapical dynactin suggest that dynein may be a candidate motor responsible for mediating transport of the nascent SV. Rotating movies of three-dimensional reconstructions of the non-treated and nocodazole-treated cells under each condition are shown in **Video S1**.

### The NVS may be a subcompartment of the Golgi or a separate compartment working sequentially with the Golgi

**Figure 3:**
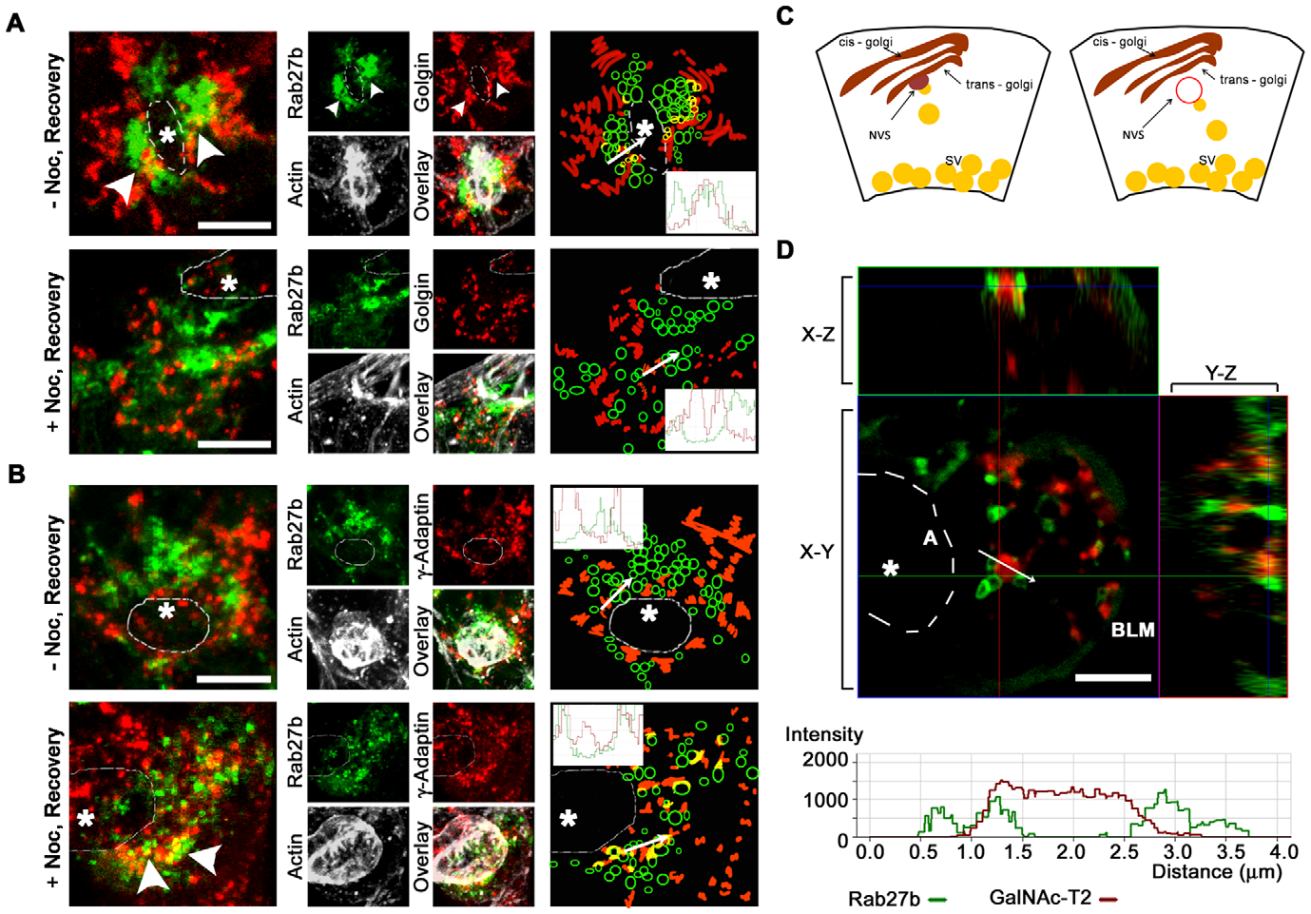
Only a subpopulation of basal YFP-Rab27b-enriched membranes partially co-localizes with late Golgi compartments. Flattened three-dimensional reconstructions of fixed LGAC show YFP-tagged Rab27b expression (green) in parallel with indirect immunofluorescence labeling of different trans-Golgi markers: **A.** Golgin 97 (*Golgin*) (N = 4) and **B.** γ-Adaptin (N = 4). For orientation, overlay images showing co-expression of Rab27b with the trans-Golgi markers are paired with those depicting actin. A schematic at the end of each row offers an interpretation of the expression patterns of the two markers, while an intensity plot of the area marked by the white arrow demonstrates co-localization patterns. Luminal regions (*) were deduced by rotation of three-dimensional stacks and determination of subapical actin orientation around this point, and *dashed lines* delineate the approximate actin accumulation in the mid-section of stack. *Arrowheads* point to regions of co-localization between Golgi markers and YFP-Rab27b. Bar represents 5 µm. **C.** A schematic of two possible origins of the budding SV suggest its association with a late compartment of the Golgi or from a physically separate NVS compartment close to the Golgi stacks. **D.** Live cell z-stack reconstruction of LGAC doubly transduced with YFP-tagged Rab27b and RFP-tagged GalNAc-T2 show x-z and y-z expression profiles. An intensity plot demonstrates the absence of overlap between the two markers. * represents lumens, *A* represents apical membrane, *BLM* represents basolateral membrane, bars represent 5 µm, N = 3.

Secreted proteins are synthesized in the ER and modified in the Golgi stacks before exiting the trans-Golgi (TGN) compartments, although these mechanisms are complex and not completely understood in acinar cells [51], [52]. Accordingly, we tested whether the NVS, the site where nocodazole treatment trapped budding SV, could be labeled with markers for the Golgi or TGN. Antibodies to golgin97, a large peripheral membrane protein with a C-terminal GRIP domain localized to the TGN [53], and γ-adaptin, a subunit of the adapter protein complex involved in late-Golgi or TGN clathrin-mediated trafficking [54] showed largely disparate labeling patterns with only a small amount of regional co-localization (<20%) which is highlighted, in the representative images in **Figure 3**, with line scans across selected areas. These regional co-localization patterns with YFP-Rab27b, however, demonstrated modest possible differences in nocodazole-treated and non-treated cells during the recovery phase from CCH stimulation. In non-treated cells, golgin97 showed some regions of co-localization with YFP-Rab27b (*arrowheads*) whereas γ-adaptin showed little or no co-localization; in nocodazole-treated cells the reverse was true (**Figure 3A, B**). These data suggested that each TGN marker, with its different co-localization patterns, might represent distinct sub-domains in the TGN, as has been previously observed for other Golgi markers [**55**].

The apparent changes in patterns of regional co-localization with either TGN marker might also reflect features related to the sequential timing of YFP-Rab27b association with different Golgi or TGN markers. For instance, the depolymerization of microtubules may sequester nascent YFP-Rab27b-enriched SV at a final fission step of SV formation, just as γ-adaptin is recruited for clathrin-mediated SV fission. The idea that nascent SV might be trapped at the moment of γ-adaptin recruitment as opposed to golgin97 recruitment was substantiated by increased regional co-localization between Rab27b and γ-adaptin in nocodazole-treated cells compared to non-treated cells. However, a complicating factor in this interpretation is that nocodazole has been observed to fragment Golgi membranes [50]. Although this effect did not appear profound in these studies, Golgi membrane fragmentation might hinder the biogenesis of nascent SV and affect the signal localization of Rab27b. A second observation worth discussing is that despite the close spatial proximity between the TGN markers and Rab27b, as previously described, the total co-localization between these markers was quite low. This was expected since at the moment the images were acquired, only a small number of the total SVs may be captured at the moment of release from the biosynthetic pathway.

While the low apparent total co-localization between Rab27b and the Golgi and/or TGN does not eliminate the possibility that these compartments are the site of origin, there are several possible explanations for the role of the NVS (**Figure 3C**): it may represent an attached compartment of the TGN, but due to the transience of the budding event, few intermediates (represented by co-localized signals) are captured by microscopy, or alternatively, the NVS is actually a distinct structure that is largely free of TGN markers. While the latter is possible, we did not find any matching descriptions of TGN-like organelles in current literature. A third scenario cannot also be ruled out- that this so-called NVS is an immature SV. The large size of the apparent NVS (2–4 µm) seems contrary to the current model of vesicle biogenesis in which small, early vesicles undergo compound fusion to increase in size [56]. Until such time that selective markers can be identified for immature SV in acinar cells, we will not be able to refute this possibility. We also tested for the co-localization of TGN46, GGA, and M6PR with Rab27b under similar conditions, but their labeling in LGAC was very weak compared to γ-adaptin and golgin97.

As an alternative, real-time imaging provided further information on the association between Rab27b-enriched SV and the Golgi and TGN. In **Figure 3D**, we examined the localization of YFP-Rab27b-enriched SV relative to a label for the late Golgi stack and TGN, by exogenously expressing the RFP-tagged N-acetylgalactosaminyltransferase 2 protein (GalNAc-T2) [57], [58]. Nocodazole-treated live cells also revealed a close association, but not a direct co-localization, between YFP-Rab27b and RFP-GalNAc-T2. An intensity profile corresponding to two YFP-Rab27b-enriched SV adjacent to a GalNAc-T2 structure (*white arrow*, *graph)* showed that Rab27b did not co-localize with GalNAc-T2 on the surface of the Golgi and appeared to label a distinct membrane compartment. This data confirmed that YFP-Rab27b-enriched nascent SV were formed in close proximity to the Golgi and/or TGN, and that YFP-Rab27b recruitment appeared to largely occur after SV formation and budding from the Golgi. We also observed that the NVS structure also was more clearly detectable in live cells compared to fixed cells, possibly due to NVS fragility and loss of its structure during the dehydration process required for cell fixation. These observations will perhaps lead to better understanding of the nature of the NVS. However, we could not unequivocally show that the NVS and the Golgi were not parts of the same structure, as suggested in the first scenario described in **Figure 3C**. It is possible that co-localization may occur but transiently, thus decreasing the likelihood of capture in real time.

### M5C associates with Rab27b on subapical SV

**Figure 4:**
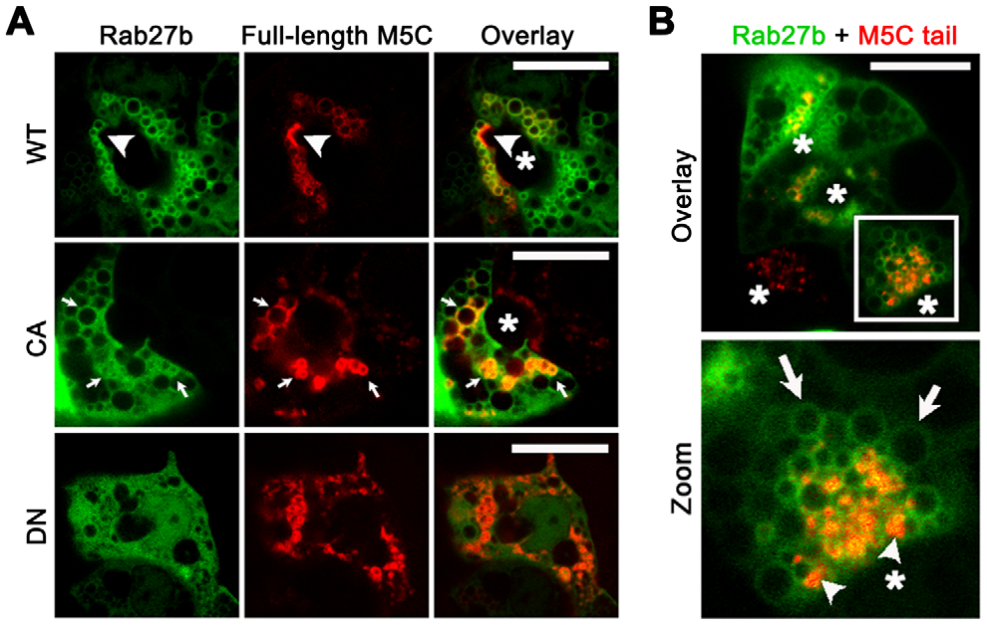
YFP-Rab27b co-localizes highly with M5C in the apical-most region of the LGAC. **A.** LGAC were co-transduced with YFP-Rab27b constructs (wild-type, constitutively-active, or dominant-negative) (*green*) and the full-length functional transcript for GFP-M5C (*red*). Live acinar cells expressing both constructs were imaged using the Zeiss Online Fingerprinting to distinguish between YFP and GFP spectra. The specific apical-most co-localization of markers is labeled by a white *arrowhead*, general co-localization is labeled by small *white arrows*. *, lumen. Bar is 10 µm, N = 4 for each construct. **B.** M5C tail, which binds the M5C site on SV but lacks the head domain to interact with actin filaments, was co-transduced with YFP-Rab27b into LGAC. In doubly-transduced LGAC, larger accumulations of Rab27b-enriched (*green, arrows*) SV appeared in some subapical regions of the acini (*inset*). M5C tail (*red, arrowheads*) was expressed as punctate labels on the membrane of SV. *, lumen. Bar is 10 µm, N = 6.

In LGAC, both M5C and Rab27b are expressed on subapical SV pools. Previous studies showed that in resting acini, M5C facilitates apical SV exocytosis, and partially co-localizes with endogenous Rab27b [6], [25], [41]. In other professional secretory cells such as melanocytes, other members of the same families of proteins have also been shown to interact with each other and with cytoskeletal proteins during intracellular trafficking [59], [60], [61], [62], [63]. Based on these studies, we speculated that M5C was a motor protein involved in the “switchover” of Rab27b-enriched SV from microtubule-based to actin-based transport. To investigate the interaction between Rab27b and M5C in real time, we conducted live cell imaging studies of LGAC transduced with adenoviral constructs to co-express the YFP-tagged wild-type or mutant Rab27b with a GFP-tagged full-length M5C (**Figure 4A**). Cells expressing both tagged proteins were most highly co-localized on the apical-most SV. We also observed that M5C localization upon SV was polarized not only by SV location, but in multiple examples was enriched on the side of the SV facing the apical plasma membrane (*large arrowhead*). This selective localization suggested that the recruitment of M5C to Rab27b-enriched SV may orient these SV to interact with the dense actin network beneath the apical plasma membrane, thus promoting the final fusion and release process.

We examined the effect of mutant Rab27b on M5C expression in order to determine whether their functions were interdependent. Previous studies showed that endogenous Rab27b and M5C in fixed LGAC were co-localized [41]. For the purposes of this study, we examined fluorescence signals in live cells between wild-type YFP-Rab27b and wild-type GFP-M5C. Measurements of the two fluorophores showed a corresponding co-localization coefficient of 55.2%±8.1% (N = 4), where wild-type YFP-Rab27b was almost entirely localized on subapical SV in resting acini. Constitutively-active YFP-Rab27b produced a strong subapical signal on SV but also a signal in the basal regions of the cell. In cells expressing the constitutively-active Rab27b protein, we found that M5C expression was still largely enriched on vesicular structures (**Figure 4A**). However, measurements of co-localization coefficients in these cells for these two proteins decreased significantly to 40.5%±3.0% (N = 4, *P*<0.05), possibly related to the increased presence of some cytosolic constitutively-active YFP-Rab27b apparent in the images. Interestingly, while co-localization remained high in the subapical region, M5C expression was not limited to SV localized directly beneath the apical membrane (*arrows*). This was particularly noticeable when comparing, in the same sample image, the distribution of M5C in the cell co-expressing the constitutively-active YFP-Rab27b versus its distribution in the adjacent cell expressing only M5C. Constitutively-active YFP-Rab27b expression resulted in an overproduction of SV in the subapical region and, possibly because of the active-locked state of Rab27b, appeared to effectively recruit M5C to the surface of Rab27b-enriched SV even before they reached the apical membrane. In cells co-expressing the dominant-negative construct, YFP-Rab27b was dispersed throughout the cytoplasm. These acini appeared depolarized and often exhibited loss of an obvious lumen, although tests showed that cell viability was not affected (data not shown). Under these conditions, M5C appeared to heavily label SV-like structures which showed minimal co-localization with the dominant-negative protein. This observation was supported by a co-localization coefficient of 28.5%±6.6%, which was significantly decreased compared to wild-type (N = 4, *P*<0.05). The partial recruitment of M5C to the surface of these non-apical SV-like structures suggested that M5C might be recruited to SV membranes independent of YFP-Rab27b recruitment and that at least a subpool of SV can form despite Rab27b dysfunction. Furthermore, while M5C was retained on a subpopulation of SV, these cells displayed a loss of polarity and expressed M5C-enriched vesicles which were not apically-localized. An alternative explanation is that M5C is associated with two vesicle pools, a Rab27b-positive subpopulation in which Rab27b regulates the recruitment of M5C, and a Rab27b-negative subpopulation. The expression of dominant-negative Rab27b might shift the preferential recruitment of M5C from the Rab27b-positive to the Rab27b-negative subpopulation. Although less likely, we also take into consideration that low level expression of endogenous Rab27b may be sufficient to induce the recruitment of M5C to SV despite the expression of excess mutant forms.

In the reverse situation, **Figure 4B** highlights an acinus co-transduced with YFP-tagged wild-type Rab27b and GFP-tagged dominant-negative M5C (M5C tail). The M5C tail construct consists of the putative cargo interaction site of the tail domain of M5C, without the actin-binding domain, and was first characterized in HeLa cells [25]. In LGAC, M5C tail expression reduced secretion of an exogenously expressed secretory protein, syncollin, suggesting it played a functional role in the acinar secretory process [6]. In co-transduced cells, M5C tail appeared highly concentrated in punctate spots or aggregates in regions localized to the surface of subapical SV, similar to the large puncta or clumps described in MCF-7 cells [25]. Interestingly enough, YFP-Rab27b localization did not appear to be obviously altered in LGAC expressing the GFP-tagged M5C tail, although the number of subapical YFP-Rab27b-enriched SV appeared particularly abundant and smaller than typically seen. This suggested that uncoupling one of the major actin-dependent motor proteins from the actin filament network through use of the dominant-negative construct did not affect the ability of Rab27b-enriched SV to be transported to the subapical region, but that it might affect their maturation (e.g., expansion) through homotypic fusion or other processes once they are in this region, resulting in an accumulation of less “mature” SV of smaller size. Although YFP-Rab27b and M5C tail expression on SV seemed to be co-localized comparably to the WT forms of these protein (39.9%±6.1%, N = 4), co-immunoprecipitation experiments in LGAC lysates of cells overexpressing wild-type Rab27b and M5C did not indicate any direct binding (data not shown). Our studies here suggest that after microtubule-dependent transport to the subapical region, recruitment of additional effectors to Rab27-enriched SV such as those described in melanosomal studies [64] may be needed to coordinate a handoff of Rab27b-enriched SV from microtubules to actin. These results represent a first glimpse at whether M5C dysfunction alone can affect Rab27b localization, since it is possible that other isoforms of M5C [6] or other effector proteins present in acinar cells may be sufficient to provide functional compensation for M5C. If M5C is recruited to both Rab27b-positive and Rab27b-negative SV, the tail of M5C may also be locked on the Rab27b-negative subpopulation and unable to be recruited to the Rab27b-positive vesicles. Future work focusing on the recruitment of additional effectors, which may interact with both Rab27b and M5C, may better elucidate the association between the two proteins.

**Figure 5:**
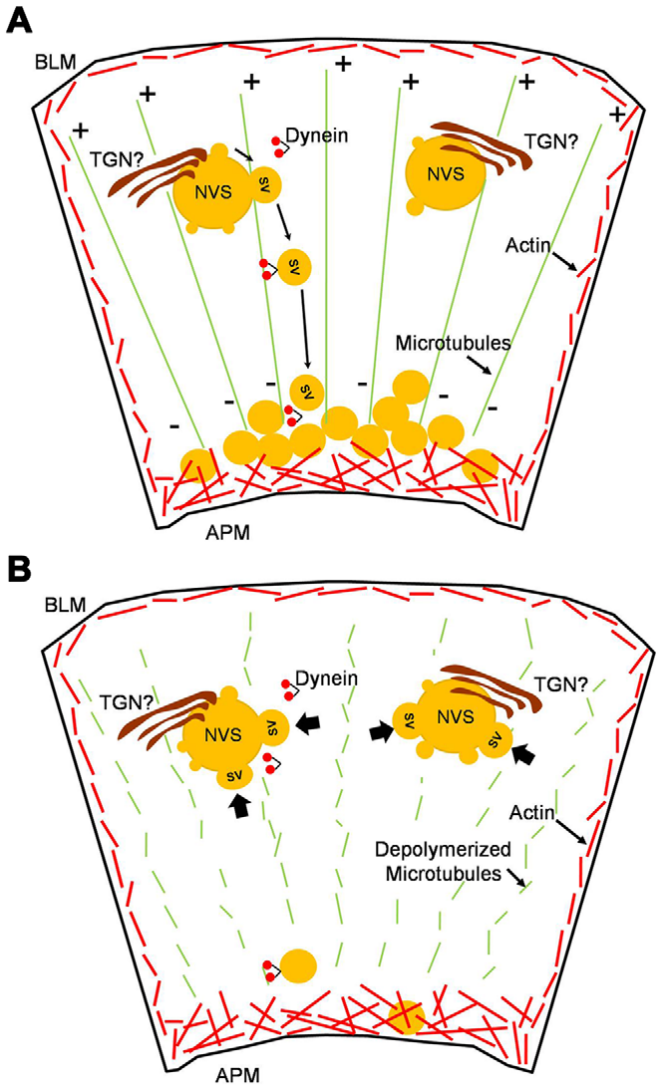
Schematic model depicting nocodazole's effect on nascent Rab27b-enriched SV formation in LGAC. **A.** Rab27b-enriched nascent SV bud from the NVS which may be a part of or associated with the TGN. Rab27b-enriched SV undergo microtubule-based transport to the subapical membrane where they join a large subapical pool awaiting stimulation for fusion with apical plasma membrane. **B.** After depolymerization of microtubules, subapical SV still undergo fusion with the apical membrane and release of content as in non-treated cells. However, newly forming nascent SV appear trapped on the NVS; SV buds are more obvious on the NVS and limited restoration of the subapical pool of SV is seen after CCH-induced depletion of the existing pool.

In summary, Rab27b enrichment begins on a nascent SV population formed at a biosynthetic compartment we termed the NVS and remains enriched throughout the process of transport and SV maturation in the subapical region. In the schematic of a LGAC at resting phase shown in **Figure 5**, nascent Rab27b-enriched SV bud from either a late Golgi compartment or a separate NVS site adjacent to or derived from the Golgi and move towards the subapical region where they accumulate in a large resting SV pool. We demonstrate in primary LGAC that Rab27b is located in close proximity to the TGN and is clearly associated with the exocytotic pathway, but is not necessarily budding from an NVS site co-localized with the TGN, or at least with the specific markers used for this study. The disruption of microtubules results in the sequestration of nascent SV on the NVS, thus limiting the repletion of the subapical SV pool. While depolymerization of microtubules affects the replenishment of a new subapical vesicle pool, it does not appear to affect the exocytosis of mature SV pools already lying beneath the apical membrane, suggesting that the role of microtubules in the maturation of SV terminates once these SV reach the subapical actin-enriched region where studies have suggested that they acquire actin binding proteins like M5C and interact with actin filaments for terminal fusion events [5], [6]. These findings emphasize the utility of live cell imaging to elucidate sequential steps in SV maturation, given the availability of an appropriate marker protein that remains associated with SV for the duration of this process.

## Materials and Methods

### Reagents

For cell culture, Matrigel™ was obtained from Collaborative Biochemicals (Bedford, MA), carbachol (Carbamoylcholine chloride) and nocodazole (Methyl-(5-[2-Thienylcarbonyl]-1H-Benzimidazol-2-YL) Carbamate) from Sigma-Aldrich (St. Louis, MO). Antibodies for immunofluorescence detection of Rab27b (mouse polyclonal B01P, Littleton, CO) were from Novus Biologicals; antibodies to p150^Glued^ (mouse monoclonal) and γ-Adaptin (mouse monoclonal) were from BD Transduction Laboratories (San Jose, CA); and antibodies to Golgin 97 (mouse monoclonal CDF4) as well as secondary antibodies and fluorescence affinity probes for microscopy: Alexa Fluor 568 donkey anti-mouse IgG, Alexa Fluor 647 phalloidin, and DAPI, were from Molecular Probes (Invitrogen, Eugene, OR). Reagents for the protease inhibitor cocktail prepared as previously described (pepstatin A, leupeptin, soybean trypsin inhibitor, tosylphenylalanylchloromethylketone, tosyllysylchloromethylketone, and phenylmethanesulphanyl fluoride) were from Sigma (St. Louis, MO) [65]. All other chemicals were reagent grade and obtained from standard suppliers.

### Primary rabbit LGAC culture

All animal procedures were in accordance with the *Guide for the Care and Use of Laboratory Animals* (NIH Publication No. 85-23, Revised 1996) [18], [66], [67] and protocols used in this study were approved by the University of Southern California IACUC approval ID 10547. LGAC from female New Zealand white rabbits (1.8–2.2 kg, Irish Farms, Norco, CA) were isolated and sequentially cultured in serum-free culture media supplemented with 0.1 µM carbachol, 1 nM thyroxine, and 5 µg/ml laminin [18], [67]. Cells were seeded on a Matrigel™ coat for fixed cell imaging on glass slips placed in 6-well plates at 2×10^6^ cells per well, or for live cell imaging on 35 mm glass coverslip bottom imaging dishes (MatTek, Ashland, MA) at 6×10^6^ cells per dish. Isolated acinar cells in culture form reconstituted acinus-like structures by day 2 of culture. These cells structurally and functionally mimic LGAC in vivo [18]. Cultured acinar cells were stimulated to secrete with CCH (100 µM, 15 m). For nocodazole treatments, cultured cells were incubated on ice for 5 m to induce depolymerization of microtubules prior to the addition of 33 µM nocodazole for 60 m at 37°C as previously described [68]. For subapical SV recovery studies, acinar cells were stimulated with CCH and then washed 4 times with warm culture media before incubation in fresh media re-perfused with nocodazole.

### Amplification and use of recombinant viruses

Replication-deficient adenovirus constructs were used to express fluorescently tagged wild-type and mutant forms of YFP-Rab27b and M5C. Wild-type exogenous YFP-Rab27b expression patterns were found, in live cell imaging studies, to be similar to endogenous protein but with increased signals. For live cell and fixed cell detection of Rab27b, constructs encoding YFP fused to the N-terminus of one of the following were expressed: Rab27b full-length (wild-type Rab27b), Rab27bQ78L (constitutively-active Rab27b), and Rab27bN133I (dominant-negative Rab27b), each generated as previously described [69]. Cell viability after transduction with Rab27b constructs was tested using the LIVE/DEAD® Cell Viability Assay Kit for mammalian cells (Invitrogen, Carlsbad, MA). For M5C studies, GFP fused to full-length human myosin 5C (wild-type M5C) and dominant-negative tail of myosin 5C (M5C tail) were prepared as described [6], [70]. Amplification of virus constructs was conducted in QBI-HEK cells (Qbiogene, Adenovirus Technology) and, upon display of cytopathological effect, supernatant was collected and purified using CsCl gradient ultracentrifugation as previously described [9]. Viral titers were measured by viral plaque formation in sequential dilutions. For optimal imaging, culture acinar cells were transduced at a MOI of 4–6 and then incubated for an additional 18–24 hours while the protein was expressed, allowing for single transduction efficiencies approximating 70–80% [5], [71] and co-transduction efficiencies approximating 50–60% per expressed protein.

Expression of RFP-tagged late Golgi/TGN marker, N-acetylgalactosaminyltransferase 2, was according to the manufacturer's instructions as specified for the use of a commercial kit, Bacmam 2.0 Cell Light Golgi (Invitrogen, Carlsbad, CA).

### Confocal fluorescence microscopy

For fixed cell and multi-channel images, cultured acinar cells were ethanol fixed and blocked with 1% BSA prior to incubation with the appropriate primary and secondary antibodies [9]. Images were collected by a Zeiss LSM 510 Meta NLO imaging system (Thornwood, NY) and translated to tiff files by Adobe Photoshop 7.0 (Adobe Systems Inc., Mountain View, CA). For live cell images and time-lapse series, cells were transduced with YFP-, GFP-, or RFP-tagged constructs and imaged in a closed, 37°C controlled airflow chamber for confocal fluorescence and DIC microscopy. For imaging of cells expressing both YFP- and GFP-tagged constructs, the Zeiss LSM 510 META Emission Fingerprinting function allowed for the separation of the two spectra as Lambda Stacks, which were then processed into a digital separation of fluorescence emissions. These images were taken in parallel with non-colocalizing samples. More detailed images were taken by z-stacks, which were reconstructed into a three-dimensional image by the Zeiss LSM Projection Tool. Unless otherwise noted, all imaging samples, both fixed and live, are representative data from repeats of at least 4 separate culture preparations (N≥4) of LGAC, harvested from different rabbits on different days and cultured at a cell density of 2×10^6^ cells per 35 mm petri dish.

For quantitative analysis of the general fluorescence intensities at the described time points, the Zeiss LSM 510 Histogram Tool was used to measure the mean intensity of the subapical region of structurally similar lumens. Fluorescence was measured in acinar clusters from at least 3 random fields per sample, where the samples were from 4 repeats of separate LGAC cultures. This subapical region was defined as an ROI with a radius of approximately half of the radius of the total area of the cells forming the lumen. The subapical fluorescent intensity was then recorded as a percentage of the total intensity within the total area of the cells. Data analysis was conducted to compare between multiple data sets using one-way ANOVA, followed by posthoc analysis using the Tukey's Post Hoc Test (PHAST TM, by Phillip R. Stanwood, Copyright 2007). The criterion for significance was *P*<0.05.

Measurement of cellular co-localization coefficients associated with distinct chromophores linked to proteins of interest were conducted using the Zeiss Enhanced Colocalization Tool software in parallel with non-colocalizing specimen. Measurements were made from the entire area of sample cells of similar surface areas. For statistical analysis, co-localization coefficients were collected from 3 random fields per sample, where the samples were from 4 repeats of separate LGAC cultures. When appropriate, data analysis was conducted to compare between data sets using Student's two-sample t-test. The criterion for significance was *P*<0.05.

## Supporting Information

Video S1 **Rotating three-dimensional reconstructions of LGAC with and without nocodazole treatment, at different phases of CCH stimulation.** LGAC expressing YFP-Rab27b are shown, comparing the effects of no treatment to nocodazole treatment on the subapical SV pool within representative acini by acquisition of z-stacks images under different conditions. Control and treated acini were imaged at resting phase prior to any stimulation, imaged after 15 m stimulation with 100 µM CCH, and then also imaged after 15 m CCH stimulation followed by multiple washes with fresh media, and 60 m recovery time without or with additional nocodazole. Results are typical of those from N = 5 separate preparations. Bar represents 5 µm.
(MP4)

Video S2 **Time-lapse video of SV pool recovery after CCH stimulation.** LGAC expressing YFP-Rab27b was stimulated with 100 µM CCH for 15 m, followed by multiple washes with fresh media. Recovery of the SV pool was imaged for 60 m. Frame rate is approximately 270∶1. Results are typical of those from N = 4 separate preparations. Bar represents 5 µm.
(MP4)

Video S3 **Time-lapse video of SV pool recovery after CCH stimulation, with nocodazole.** After treatment with nocodazole, LGAC expressing YFP-Rab27b was stimulated with 100 µM CCH for 15 m, followed by multiple washes with fresh media. Recovery of the SV pool was imaged for the following 60 m. Frame rate is approximately 200∶1. Results are typical of those from N = 5 separate preparations. Bar represents 5 µm.
(MP4)

## References

[1] Fullard RJ, Snyder C (1990). Protein levels in nonstimulated and stimulated tears of normal human subjects.. Invest Ophthalmol Vis Sci.

[2] de Souza GA, Godoy LM, Mann M (2006). Identification of 491 proteins in the tear fluid proteome reveals a large number of proteases and protease inhibitors.. Genome Biol.

[3] Sanghi S, Kumar R, Lumsden A, Dickinson D, Klepeis V (2001). cDNA and genomic cloning of lacritin, a novel secretion enhancing factor from the human lacrimal gland.. J Mol Biol.

[4] van Setten GB, Viinikka L, Tervo T, Pesonen K, Tarkkanen A (1989). Epidermal growth factor is a constant component of normal human tear fluid.. Graefes Arch Clin Exp Ophthalmol.

[5] Jerdeva GV, Wu K, Yarber FA, Rhodes CJ, Kalman D (2005a). Actin and non-muscle myosin II facilitate apical exocytosis of tear proteins in rabbit lacrimal acinar epithelial cells.. J Cell Sci.

[6] Marchelletta RR, Jacobs DT, Schechter JE, Cheney RE, Hamm-Alvarez SF (2008). The Class V myosin motor, Myosin 5c, localizes to mature secretory vesicles and facilitates exocytosis in lacrimal acini.. Am J Physiol Cell Physiol.

[7] Jedd G, Richardson C, Litt R, Segev N (1995). The Ypt1 GTPase is essential for the first two steps of the yeast secretory pathway.. J Cell Biol.

[8] Baker D, Wuestehube L, Schekman R, Botstein D, Segev N (1990). GTP-binding Ypt1 protein and Ca2+ function independently in a cell-free protein transport reaction.. Proc Natl Acad Sci U S A.

[9] Wang Y, Jerdeva G, Yarber FA, da Costa SR, Xie J (2003). Cytoplasmic dynein participates in apically targeted stimulated secretory traffic in primary rabbit lacrimal acinar epithelial cells.. J Cell Sci.

[10] Valentijn JA, Valentijn K, Pastore LM, Jamieson JD (2000). Actin coating of secretory granules during regulated exocytosis correlates with the release of rab3D.. Proc Natl Acad Sci U S A.

[11] Yoshie S, Imai A, Nashida T, Shimomura H (2000). Expression, characterization, and localization of Rab26, a low molecular weight GTP-binding protein, in the rat parotid gland.. Histochem Cell Biol.

[12] Pickett JA, Edwardson JM (2006). Compound exocytosis: mechanisms and functional significance.. Traffic.

[13] Apodaca G (2001). Endocytic traffic in polarized epithelial cells: role of the actin and microtubule cytoskeleton.. Traffic.

[14] Valentijn KM, Gumkowski FD, Jamieson JD (1999). The subapical actin cytoskeleton regulates secretion and membrane retrieval in pancreatic acinar cells.. J Cell Sci.

[15] D'Souza-Schorey C, Chavrier P (2006). ARF proteins: roles in membrane traffic and beyond.. Nat Rev Mol Cell Biol.

[16] Davidson HW, Balch WE (1993). Differential inhibition of multiple vesicular transport steps between the endoplasmic reticulum and trans Golgi network.. J Biol Chem.

[17] Miserey-Lenkei S, Chalancon G, Bardin S, Formstecher E, Goud B Rab and actomyosin-dependent fission of transport vesicles at the Golgi complex.. Nat Cell Biol.

[18] da Costa SR, Yarber FA, Zhang L, Sonee M, Hamm-Alvarez SF (1998). Microtubules facilitate the stimulated secretion of beta-hexosaminidase in lacrimal acinar cells.. J Cell Sci.

[19] Fath KR, Trimbur GM, Burgess DR (1994). Molecular motors are differentially distributed on Golgi membranes from polarized epithelial cells.. J Cell Biol.

[20] Wu K, Jerdeva GV, da Costa SR, Sou E, Schechter JE (2006). Molecular mechanisms of lacrimal acinar secretory vesicle exocytosis.. Exp Eye Res.

[21] Wu X, Bowers B, Wei Q, Kocher B, Hammer JA (1997). Myosin V associates with melanosomes in mouse melanocytes: evidence that myosin V is an organelle motor.. J Cell Sci.

[22] Lapierre LA, Goldenring JR (2005). Interactions of myosin vb with rab11 family members and cargoes traversing the plasma membrane recycling system.. Methods Enzymol.

[23] Lise MF, Wong TP, Trinh A, Hines RM, Liu L (2006). Involvement of myosin Vb in glutamate receptor trafficking.. J Biol Chem.

[24] Nedvetsky PI, Stefan E, Frische S, Santamaria K, Wiesner B (2007). A Role of myosin Vb and Rab11-FIP2 in the aquaporin-2 shuttle.. Traffic.

[25] Jacobs DT, Weigert R, Grode KD, Donaldson JG, Cheney RE (2009). Myosin Vc is a molecular motor that functions in secretory granule trafficking.. Mol Biol Cell.

[26] Pereira-Leal JB, Seabra MC (2001). Evolution of the Rab family of small GTP-binding proteins.. J Mol Biol.

[27] Seabra MC, Mules EH, Hume AN (2002). Rab GTPases, intracellular traffic and disease.. Trends Mol Med.

[28] Wu H, Rossi G, Brennwald P (2008). The ghost in the machine: small GTPases as spatial regulators of exocytosis.. Trends Cell Biol.

[29] Seabra MC, Coudrier E (2004). Rab GTPases and myosin motors in organelle motility.. Traffic.

[30] Grosshans BL, Ortiz D, Novick P (2006). Rabs and their effectors: achieving specificity in membrane traffic.. Proc Natl Acad Sci U S A.

[31] Schluter OM, Khvotchev M, Jahn R, Sudhof TC (2002). Localization versus function of Rab3 proteins. Evidence for a common regulatory role in controlling fusion.. J Biol Chem.

[32] Evans E, Zhang W, Jerdeva G, Chen CY, Chen X (2008). Direct interaction between Rab3D and the polymeric immunoglobulin receptor and trafficking through regulated secretory vesicles in lacrimal gland acinar cells.. Am J Physiol Cell Physiol.

[33] Riedel D, Antonin W, Fernandez-Chacon R, Alvarez de Toledo G, Jo T (2002). Rab3D is not required for exocrine exocytosis but for maintenance of normally sized secretory granules.. Mol Cell Biol.

[34] Chen X, Edwards JA, Logsdon CD, Ernst SA, Williams JA (2002). Dominant negative Rab3D inhibits amylase release from mouse pancreatic acini.. J Biol Chem.

[35] Nguyen D, Jones A, Ojakian GK, Raffaniello RD (2003). Rab3D redistribution and function in rat parotid acini.. J Cell Physiol.

[36] Ostrowski M, Carmo NB, Krumeich S, Fanget I, Raposo G (2010). Rab27a and Rab27b control different steps of the exosome secretion pathway.. Nat Cell Biol.

[37] Chen D, Guo J, Miki T, Tachibana M, Gahl WA (1997). Molecular cloning and characterization of rab27a and rab27b, novel human rab proteins shared by melanocytes and platelets.. Biochem Mol Med.

[38] Chen X, Li C, Izumi T, Ernst SA, Andrews PC (2004). Rab27b localizes to zymogen granules and regulates pancreatic acinar exocytosis.. Biochem Biophys Res Commun.

[39] Imai A, Yoshie S, Nashida T, Shimomura H, Fukuda M (2004). The small GTPase Rab27B regulates amylase release from rat parotid acinar cells.. J Cell Sci.

[40] Imai A, Yoshie S, Nashida T, Fukuda M, Shimomura H (2009). Redistribution of small GTP-binding protein, Rab27B, in rat parotid acinar cells after stimulation with isoproterenol.. Eur J Oral Sci.

[41] Chiang L, Ngo J, Schechter JE, Karvar S, Tolmachova T (2011). Rab27b regulates exocytosis of secretory vesicles in acinar epithelial cells from the lacrimal gland.. Am J Physiol Cell Physiol.

[42] Tolmachova T, Abrink M, Futter CE, Authi KS, Seabra MC (2007). Rab27b regulates number and secretion of platelet dense granules.. Proc Natl Acad Sci U S A.

[43] Gomi H, Mori K, Itohara S, Izumi T (2007). Rab27b is expressed in a wide range of exocytic cells and involved in the delivery of secretory granules near the plasma membrane.. Mol Biol Cell.

[44] Jerdeva GV, Yarber FA, Trousdale MD, Rhodes CJ, Okamoto CT (2005b). Dominant-negative PKC-epsilon impairs apical actin remodeling in parallel with inhibition of carbachol-stimulated secretion in rabbit lacrimal acini.. Am J Physiol Cell Physiol.

[45] Kraemer J, Schmitz F, Drenckhahn D (1999). Cytoplasmic dynein and dynactin as likely candidates for microtubule-dependent apical targeting of pancreatic zymogen granules.. Eur J Cell Biol.

[46] Vaughan KT (2005). Microtubule plus ends, motors, and traffic of Golgi membranes.. Biochim Biophys Acta.

[47] Gill SR, Schroer TA, Szilak I, Steuer ER, Sheetz MP (1991). Dynactin, a conserved, ubiquitously expressed component of an activator of vesicle motility mediated by cytoplasmic dynein.. J Cell Biol.

[48] Qian L, Xie J, Rose CM, Sou E, Zeng H (2004). Altered traffic to the lysosome in an ex vivo lacrimal acinar cell model for chronic muscarinic receptor stimulation.. Exp Eye Res.

[49] Busson-Mabillot S, Chambaut-Guerin AM, Ovtracht L, Muller P, Rossignol B (1982). Microtubules and protein secretion in rat lacrimal glands: localization of short-term effects of colchicine on the secretory process.. J Cell Biol.

[50] Robin P, Rossignol B, Raymond MN (1995). Effect of microtubule network disturbance by nocodazole and docetaxel (Taxotere) on protein secretion in rat extraorbital lacrimal and parotid glands.. Eur J Cell Biol.

[51] Goud B, Gleeson PA (2010). TGN golgins, Rabs and cytoskeleton: regulating the Golgi trafficking highways.. Trends Cell Biol.

[52] Pfeffer SR (2007). Unsolved mysteries in membrane traffic.. Annu Rev Biochem.

[53] Yoshino A, Bieler BM, Harper DC, Cowan DA, Sutterwala S (2003). A role for GRIP domain proteins and/or their ligands in structure and function of the trans Golgi network.. J Cell Sci.

[54] Wong DH, Brodsky FM (1992). 100-kD proteins of Golgi- and trans-Golgi network-associated coated vesicles have related but distinct membrane binding properties.. J Cell Biol.

[55] Derby MC, van Vliet C, Brown D, Luke MR, Lu L (2004). Mammalian GRIP domain proteins differ in their membrane binding properties and are recruited to distinct domains of the TGN.. J Cell Sci.

[56] Kogel T, Gerdes HH (2010). Maturation of secretory granules.. Results Probl Cell Differ.

[57] White T, Bennett EP, Takio K, Sorensen T, Bonding N (1995). Purification and cDNA cloning of a human UDP-N-acetyl-alpha-D-galactosamine:polypeptide N-acetylgalactosaminyltransferase.. J Biol Chem.

[58] Rottger S, White J, Wandall HH, Olivo JC, Stark A (1998). Localization of three human polypeptide GalNAc-transferases in HeLa cells suggests initiation of O-linked glycosylation throughout the Golgi apparatus.. J Cell Sci.

[59] Hume AN, Collinson LM, Rapak A, Gomes AQ, Hopkins CR (2001). Rab27a regulates the peripheral distribution of melanosomes in melanocytes.. J Cell Biol.

[60] Hume AN, Tarafder AK, Ramalho JS, Sviderskaya EV, Seabra MC (2006). A coiled-coil domain of melanophilin is essential for Myosin Va recruitment and melanosome transport in melanocytes.. Mol Biol Cell.

[61] Izumi T, Gomi H, Kasai K, Mizutani S, Torii S (2003). The roles of Rab27 and its effectors in the regulated secretory pathways.. Cell Struct Funct.

[62] Kuroda TS, Itoh T, Fukuda M (2005). Functional analysis of slac2-a/melanophilin as a linker protein between Rab27A and myosin Va in melanosome transport.. Methods Enzymol.

[63] Wu X, Rao K, Bowers MB, Copeland NG, Jenkins NA (2001). Rab27a enables myosin Va-dependent melanosome capture by recruiting the myosin to the organelle.. J Cell Sci.

[64] Fukuda M (2006). Distinct Rab27A binding affinities of Slp2-a and Slac2-a/melanophilin: Hierarchy of Rab27A effectors.. Biochem Biophys Res Commun.

[65] Vilalta PM, Zhang L, Hamm-Alvarez SF (1998). A novel taxol-induced vimentin phosphorylation and stabilization revealed by studies on stable microtubules and vimentin intermediate filaments.. J Cell Sci.

[66] Gierow JP, Yang T, Bekmezian A, Liu N, Norian JM (1996). Na-K-ATPase in lacrimal gland acinar cell endosomal system: correcting a case of mistaken identity.. Am J Physiol.

[67] Gierow JP, Lambert RW, Mircheff AK (1995). Fluid phase endocytosis by isolated rabbit lacrimal gland acinar cells.. Exp Eye Res.

[68] da Costa SR, Andersson S, Arber F, Okamoto C, Hamm-Alvarez S (2002). Cytoskeletal participation in stimulated secretion and compensatory apical plasma membrane retrieval in lacrimal gland acinar cells.. Adv Exp Med Biol.

[69] Suda J, Zhu L, Okamoto C, Karvar S (2010). (in press)) Cellular distribution and functional importance of Rab27b in gastric parietal cells.. Gastroenterology.

[70] Rodriguez OC, Cheney RE (2002). Human myosin-Vc is a novel class V myosin expressed in epithelial cells.. J Cell Sci.

[71] Xie J, Chiang L, Contreras J, Wu K, Garner JA (2006). Novel fiber-dependent entry mechanism for adenovirus serotype 5 in lacrimal acini.. J Virol.

